# 90 Efficacy of Porcine Skin Xenotransplants Indistinguishable from Allograft in First-in-human Clinical Evaluation

**DOI:** 10.1093/jbcr/irac012.093

**Published:** 2022-03-23

**Authors:** Paul Holzer, Elizabeth J Chang, Diana Lu, Jon Adkins, Kaitlyn Rogers, Angela Man, Kristina Chang, Joan Wicks, Dicken S C Ko, John T Schulz, Robert A Kaiser, Harold M Swartz, Curtis L Cetrulo, Rod Monroy, Jeremy Goverman

**Affiliations:** Johns Hopkins University, Boston, Massachusetts; XenoTherapeutics, Inc, Boston, Massachusetts; XenoTherapeutics, Inc, Boston, Massachusetts; XenoTherapeutics, Inc, Boston, Massachusetts; XenoTherapeutics, Inc, Boston, Massachusetts; Massachusetts General Hospital, Boston, Massachusetts; Massachusetts General Hospital, Boston, Massachusetts; StageBio, Frederick, Maryland; Rhode Island Hospital, The Warren Alpert Medical School of Brown University, Providence, Rhode Island; Massachusetts General Hospital, Boston, Massachusetts; Cytotheryx, Rochester, Minnesota; Dartmouth Geisel School of Medicine, Lyme, New Hampshire; Massachusetts General Hospital, Shiners Hospital for Children-Boston, Boston, Massachusetts; XenoTherapeutics, Inc, Boston, Massachusetts; Massachusetts General Hospital, Boston, Massachusetts

## Abstract

**Introduction:**

Human cadaver allograft (HCA) is the current standard of care for temporary wound closure of large burns, but a critical need for high-quality alternatives exists. Porcine skin shares many similarities with human skin, and hyperacute rejection is prevented via a single genetic modification. Like human skin, non-terminally sterilized porcine skin contains viable dermal and epidermal cells and intact vasculature that enables restoration of barrier function. These characteristics are distinct from those of traditional, terminally sterilized “xenografts” and may offer greater therapeutic capability. We report here key efficacy outcomes specific to severe burn care from a first-in-human clinical trial to assess the capability of genetically engineered porcine skin xenotransplants to provide temporary wound closure for severe and extensive deep-partial and full-thickness burn wounds, compared to HCA.

**Methods:**

Split-thickness skin containing epidermal and dermal layers was harvested from Designated Pathogen Free, GalT-KO, porcine donors, cGMP processed to achieve USP< 71 > sterility and cryopreserved to retain >70% cell viability. These were transplanted side by side with HCA on debrided full-thickness burn wounds in six human subjects. Temporary wound closure, incidence of complete wound closure following autografting, and quality of healing, including scarring, contour, and feel of healed skin, normalization of skin markings or pigmentation, were independently assessed.

**Results:**

Across all patients and assessment time points, adherence, vascularity, and overall appearance were indistinguishable between porcine skin xenotransplants and HCA control. After surgical removal, wound beds treated with each type of dressing were perfused and otherwise appeared equivalent and clinically suitable for autografting. Long-term outcomes were comparable between wound sites treated with porcine skin or HCA with no discernable differences in scarring or cosmesis.

**Conclusions:**

Skin xenotransplants effectively provided temporary wound closure and restoration of barrier function via intact native vasculature, active cells, decreased antigenicity, and high-quality tissue architecture unimpacted by cryopreservation and thawing. These results show clinical promise as an interchangeable alternative to HCA in the treatment of severe burns. Expanded clinical evaluation is ongoing.

